# New perspectives from evidence to action — patient-centered evidence transformation in evidence-based nursing: a narrative review

**DOI:** 10.3389/fpubh.2026.1877125

**Published:** 2026-08-28

**Authors:** Zhe-Hao Hu, Liu Han, Dan Yang, Xiao-Yan Zhang, Wan-Ting Shen, Yue-Tong Wang, Fang-Shuo Zhang, Xue-Jing Li, Yu-Fang Hao

**Affiliations:** 1 School of Nursing, Beijing University of Chinese Medicine, Beijing, China; 2 Evidence-Based Nursing Research Center, School of Nursing, Beijing University of Chinese Medicine, Beijing, China; 3 RNAO-BPSO Host Research Center, Beijing, China; 4 Beijing University of Chinese Medicine Third Affiliated Hospital, Beijing, China; 5 Fangshan Hospital Beijing University of Chinese Medicine, Beijing, China; 6 Beijing Hospital, Beijing, China

**Keywords:** evidence transformation, evidence-based nursing, Knowledge-to-Action Model, narrative review, patient decision aid, patient-centered care, RNAO - BPSO, Social Movement Model

## Abstract

**Background:**

Evidence transformation remains a major challenge in nursing practice, as existing approaches often focus on professional-driven implementation while insufficiently incorporating patient participation, accessibility of evidence, and contextual adaptation. A comprehensive patient-centered pathway for translating evidence into practice is needed.

**Aims:**

This narrative review aimed to critically examine existing evidence transformation models and develop a Patient-Centered Evidence Transformation Framework to support sustainable evidence-based nursing practice.

**Methods:**

A narrative review approach was adopted to synthesize literature on evidence transformation, implementation science, patient-centered care, and integrative nursing practice. Existing theoretical models, including the Knowledge-to-Action Model, Social Movement Model, Promoting Action on Research Implementation in Health Services Framework, Consolidated Framework for Implementation Research, and Shared Decision-Making Model, were critically analyzed. A conceptual framework was developed through theoretical integration and synthesis of patient-centered evidence transformation principles.

**Results:**

Existing models provide important insights into knowledge translation, implementation processes, and patient engagement but often address isolated components rather than a continuous transformation pathway. This review proposes a six-component Patient-Centered Evidence Transformation Framework, including Evidence Creation, Patient Version of Guidelines Development, Evidence-Based Science Popularization, Patient Decision Aids, Evidence Dissemination, and Application of Evidence. The framework highlights patients as active participants throughout the evidence lifecycle and provides guidance for evidence transformation in integrative Chinese and Western nursing practice.

**Conclusion:**

The proposed framework integrates evidence generation, patient empowerment, dissemination, and clinical application into a unified pathway.

## Graphical abstract

**Figure:**
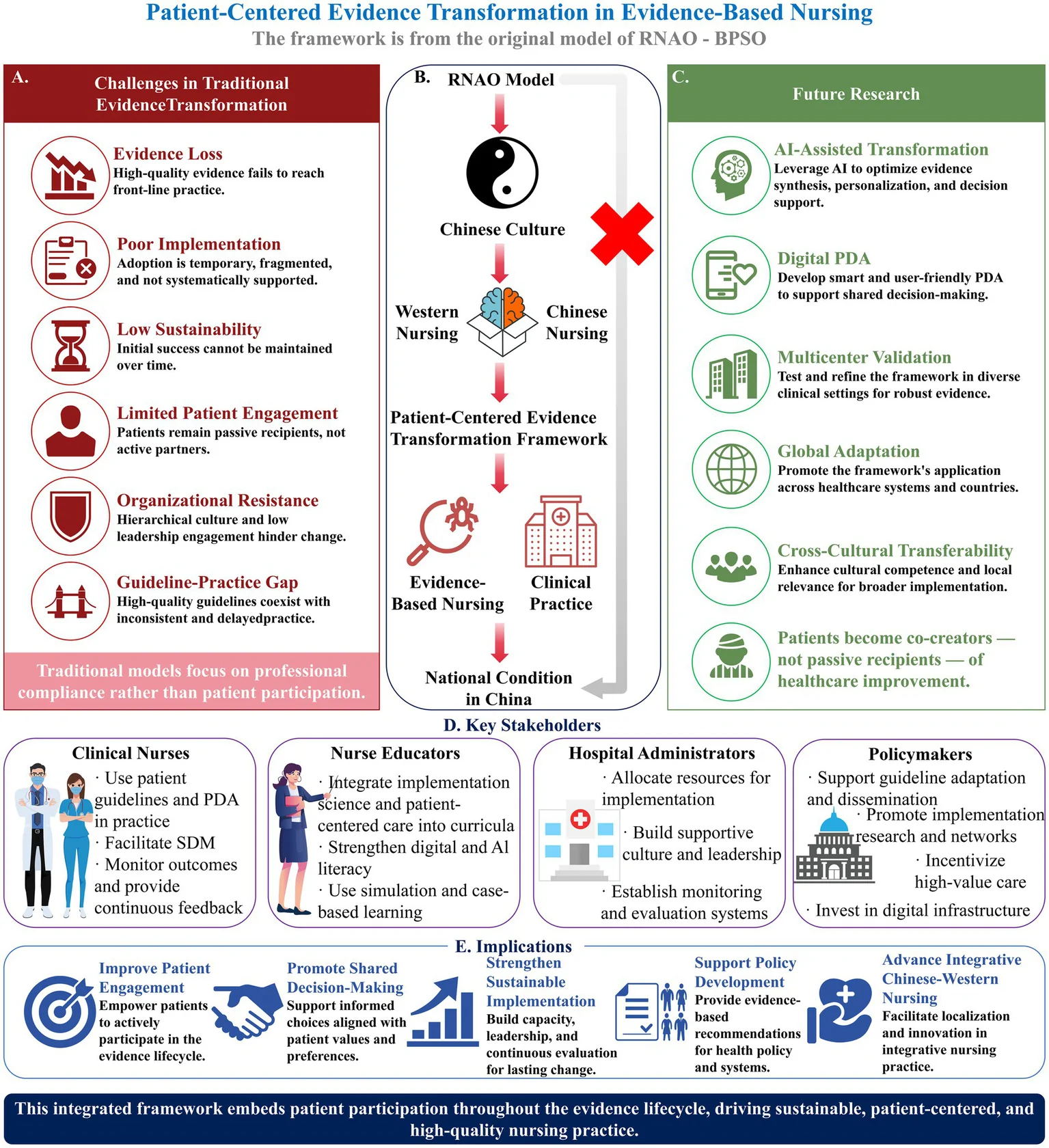
(A) Challenges in traditional evidence transformation; (B) The RNAO model cannot directly adapt to national conditions in China. First, it needs to integrate Chinese culture and generate a new framework through the interaction between Chinese and Western nursing practices. Only after years of evidence-based nursing and clinical practice can it ultimately adapt to national conditions in China; (C) Future research; (D) Key stakeholders; (E) Implications. RNAO - BPSO, Registered Nurses’ Association of Ontario–Best Practice Spotlight Organizations; KTA, Knowledge-to-Action; SMM, Social Movement Model; AI, Artificial intelligence; PDA, Patient decision aids.

## 1 Introduction

With the continuous global advancement of evidence-based nursing, transforming high-quality research evidence into safe, effective, sustainable, and easily accessible clinical services remains one of the most persistent challenges facing global healthcare systems (1). Although significant progress has been made in developing clinical practice guidelines, conducting systematic reviews, and implementing strategies, a substantial gap persists between evidence creation and its practical application in routine clinical practice (2). This “cognition-practice gap” is particularly evident in nursing practice: evidence implementation is often delayed, fragmented, or lacks continuity, leading to diminished evidence value and suboptimal patient outcomes (3).

In recent years, the patient-centered care model has become a core principle for improving healthcare quality and evidence-based practice. Unlike approaches that solely emphasize strict adherence to clinical guidelines by healthcare providers, the patient-centered transformation emphasizes patients’ active participation throughout the entire evidence lifecycle, encompassing evidence adaptation, decision-making, implementation, outcome evaluation, and continuous improvement (4). Shared Decision-Making (SDM), Patient Decision Aids (PDA), patient-friendly guidelines, and health literacy interventions have been proven effective in enhancing treatment adherence, patient satisfaction, and health outcomes (5). However, in most existing knowledge translation pathways, patient involvement remains peripheral rather than central, and implementation efforts prioritize changes in healthcare providers’ behaviors over genuine patient participation (6).

Traditional knowledge translation models provide crucial structured pathways for evidence implementation, yet they also exhibit significant limitations. Taking the “Knowledge to Action” (KTA) framework as an example, it offers a systematic roadmap for evidence generation and application, but primarily focuses on technical processes and implementation steps (3). In reality, evidence transformation is not merely a technical task; it also requires organizational cultural transformation, leadership development, stakeholder engagement, and sustained collective action. Failure to address these social and behavioral challenges often impedes the long-term sustainability of evidence implementation (7). Similarly, many implementation strategies emphasize “guideline adoption” rather than “patient-centered guideline optimization,” thereby limiting the realization of truly patient-centered healthcare models (8).

The Best Practice Guidelines developed by the Registered Nurses of Ontario (RNAO) and the Global Best Practice Organization Network (BPSO) provide an internationally recognized paradigm for bridging the gap between evidence and practice in nursing (9). This framework, built upon the RNAO’s “Change Leadership Toolkit,” integrates the structured implementation logic of the KTA model with the Social Movement Model (SMM)—which emphasizes bottom-up participation mechanisms, collective identity, intrinsic motivation, and change leadership (10). Through its dual-driven strategy of “structure + dynamics,” the BPSO model extends evidence application from individual clinical practice to organizational transformation and patient engagement, and has been successfully implemented in multiple countries and healthcare systems (11).

As shown in graphical abstract, the international community has increasingly emphasized implementation science and patient-centered care (12, 13). However, evidence transformation frameworks that systematically integrate patient participation, social mobilization, contextual adaptation, and sustainable implementation remain insufficiently developed, particularly within integrated Chinese and Western nursing. In this review, integrated Chinese and Western nursing refers to a holistic nursing approach that combines evidence-based Western nursing practices with appropriate traditional Chinese medicine (TCM)-based nursing concepts and interventions to address patients’ physical, psychological, and cultural needs through individualized care (14).

Although existing studies have explored individual strategies such as PDA, patient versions of clinical guidelines, or evidence dissemination approaches, these components are often implemented separately rather than within a comprehensive transformation pathway (11, 15). This limitation is particularly relevant to integrated Chinese and Western nursing, where effective evidence implementation requires not only clinical evidence integration but also patient engagement, cultural adaptation, and collaboration among healthcare professionals and patients (16). Therefore, there is an urgent need for a narrative review to examine current challenges in evidence transformation, analyze the strengths and limitations of existing theoretical models, and develop a more comprehensive, patient-centered, and sustainable evidence transformation framework for nursing practice.

This narrative review aims to synthesize the KTA model and the SMM, drawing on the original RNAO - BPSO framework and its implementation experiences, as well as the practical context of integrated Chinese and Western nursing, to systematically organize current evidence supporting patient-centered evidence-based practice translation. By proposing a comprehensive framework that integrates patient participation throughout the entire evidence lifecycle, this review seeks to provide a theoretical foundation and practical roadmap for the localized application of international guidelines and the high-quality development of evidence-based nursing practices.

## 2 Methods

This study adopted a narrative review approach because the purpose was to synthesize theoretical perspectives, critically examine existing evidence transformation models, and develop a patient-centered conceptual framework rather than to estimate intervention effects. Narrative reviews are appropriate for integrating diverse bodies of literature, exploring complex concepts, and generating theoretical insights when evidence is heterogeneous across disciplines (17–19). The review process was guided by established recommendations for narrative reviews and conceptual framework development, including SANRA principles and approaches for theory synthesis (20) (For details on Methods, please refer to the Supplementary materials).

## 3 Challenges in traditional evidence transformation

As shown in graphical abstract (A), traditional evidence transformation approaches often fail due to their excessive emphasis on knowledge transfer while underestimating the importance of contextual adaptability, patient engagement, organizational readiness, and long-term sustainability. As illustrated in Table 1, evidence erosion, suboptimal implementation outcomes, insufficient sustainability, low patient participation, organizational resistance, and persistent gaps between guidelines and practice collectively limit the effectiveness of evidence-based nursing practices. These challenges demonstrate that successful evidence transformation requires not only structured implementation pathways but also a patient-centered framework that supports co-creation, social mobilization, and sustainable organizational change (21, 22).

**Table 1:** The major challenges faced in the transformation of traditional evidence.

Major challenges	Main manifestations	Primary cause	Impact on evidence-based nursing
Evidence erosion	High-quality evidence has not been applied in frontline clinical practice; relevant guidelines remain confined to the academic realm and have not been translated into routine diagnostic and treatment protocols (23).	The fragmentation phenomena in evidence synthesis, dissemination, contextual adaptation, and implementation; insufficient localization of international guidelines (24).	This evidence is theoretically valid but difficult to apply in practice, thereby reducing its clinical relevance and patient-centered value (25).
Suboptimal implementation outcomes	The adoption of guidelines is characterized by its temporary and fragmented nature, typically based on specific promotional campaigns rather than systematic frameworks (26).	Excessive reliance on one-off training sessions, policy dissemination, and administrative directives; insufficient leadership support, feedback mechanisms, and resource allocation (27).	Short-term adherence was not accompanied by stable behavioral changes; the implementation fidelity was low (28).
Insufficient sustainability	The successful outcomes achieved during the initial implementation phase cannot be sustained over the long term (29).	High employee turnover rates, substantial nursing workload pressures, staff shortages, conflicting clinical priorities, and the absence of sustained monitoring mechanisms (30).	It is challenging to integrate best practices into routine care workflows and organizational culture (31).
Low patient participation	Patients remain passive recipients rather than active participants in the medical decision-making process (32).	Traditional models place greater emphasis on clinical healthcare adherence rather than patients’ comprehension ability, collaborative decision-making processes, or health literacy (33).	Reduced treatment adherence, decreased patient satisfaction, and diminished efficacy of evidence-based interventions (34).
Organizational resistance	Surface-level compliance with evidence and resistance to change (9, 35).	A hierarchical management structure, rigid professional boundaries, low psychological safety, insufficient leadership engagement, and a lack of an innovation culture (9, 35).	This implementation approach adopts a top-down approach and lacks continuity, thereby limiting the advancement of long-term transformation (35, 36).
persistent gaps between guidelines and practice	High-quality guidelines coexist with inconsistent bedside practices and delayed adoption (37).	Time constraints, insufficient institutional support, limited nursing authority, disparities in policy implementation, and local environmental barriers (38).	A persistent gap exists between evidence creation and actual clinical outcomes, particularly in integrated care settings (38, 39).

## 4 Existing theoretical models/frameworks for evidence transformation

Currently, multiple theoretical models/frameworks have been widely applied in evidence transformation and implementation within the healthcare sector, providing critical perspectives for translating research evidence into clinical practice, driving organizational change, and enhancing patient benefits. Among these, the KTA model, SMM, Promoting Action on Research Implementation in Health Services (PARIHS) framework, Consolidated Framework for Implementation Research (CFIR), and SDM model are the most representative. While each model/framework offers distinct advantages in guiding evidence implementation, they all exhibit significant limitations when applied to patient-centered evidence transformation. Most existing models/frameworks focus on structured implementation pathways, situational assessments, or patient engagement at specific stages, with few encompassing the entire evidence lifecycle—including evidence creation, dissemination, organizational mobilization, and sustained patient participation (3, 9, 36, 40). Notably, patients are often portrayed as passive recipients of evidence rather than active participants in transformative processes (2). This limitation is particularly pronounced in complex healthcare systems such as integrated Chinese and Western nursing systems, which require localized characteristics, cultural adaptability, and long-term sustainability. Therefore, there is an urgent need to develop a comprehensive framework. Table 2 provides a comparative overview of the major existing models.

**Table 2:** Comparison of existing theoretical models/frameworks for evidence transformation.

Model	Core components	Strengths	Limitations	Relevance to patient-centered evidence transformation
KTA model	The knowledge creation and action cycle encompasses evidence creation, integration, adaptation, implementation, monitoring, evaluation, and sustainability (3, 41).	A clear, structured, and iterative pathway for translating evidence into practice; highly operational (3, 41).	The focus is primarily on technical implementation and procedural rigor; attention to motivational factors and cultural change is limited (36).	It supports evidence conversion throughout the entire process, applicable to guideline development, patient version of guidelines, and PDA construction (3, 41).
SMM	Prerequisites, key characteristics, and outcomes; emphasizing urgency, leadership, collective identity, intrinsic motivation, and network relationships (36, 42).	It demonstrates strong efficacy in driving bottom-up mobilization, fostering emotional engagement, and facilitating organizational cultural transformation (36, 42).	There is a lack of standardized implementation steps and formal evidence transformation pathways (36).	It enhances the sustainability of patient-centered transformation through collective participation (36, 42).
PARIHS framework	Evidence, context, and facilitating factors collectively determine the success of implementation (43, 44).	The focus is on the organizational environment, leadership, coordination mechanisms, and patient preferences (43, 44).	The concept is vague and the operational guidance is inadequate (45).	It supports context-adaptive adjustments and patient-centered implementation (44).
CFIR	Characteristics of intervention measures, external environment, internal environment, individual characteristics, and implementation process (16).	Comprehensive identification of both obstructive and promotive factors; demonstrates strong explanatory power (16, 36).	It primarily serves a diagnostic purpose rather than a prescriptive one; it lacks a direct implementation roadmap (36, 46).	It facilitates the evaluation of the feasibility and contextual adaptability of patient-centered evidence-based practices (16, 36).
SDM model	Evidence-based, collaborative decision-making incorporating patient values and preferences; supported by PDA and patient guidelines (2, 10).	This represents the most direct manifestation of a patient-centered care philosophy; it enhances decision-making quality and patient satisfaction (10, 11).	The primary focus is on individual clinical decision-making rather than systemic-level reforms (2, 47).	It is crucial to integrate patient participation into the evidence transformation process (2, 10, 48).

KTA, Knowledge-to-Action; PDA, patient decision aids; SMM, Social Movement Model; PARIHS, Promoting Action on Research Implementation in Health Services; CFIR, Consolidated Framework for Implementation Research; SDM, shared decision-making.

## 5 The “patient-centered” evidence transformation framework

As shown in graphical abstract (B), this study proposes a “patient-centered” evidence transformation framework based on integrated Chinese and Western evidence-based nursing, building upon the original RNAO - BPSO model. Unlike traditional evidence transformation models that primarily focus on professional implementation pathways, this framework places patients at the core of the entire evidence lifecycle, emphasizing the dynamic interactions among evidence creation, patient empowerment, organizational mobilization, and sustainable practice transformation (5, 42, 49, 50).

**Figure 1:**
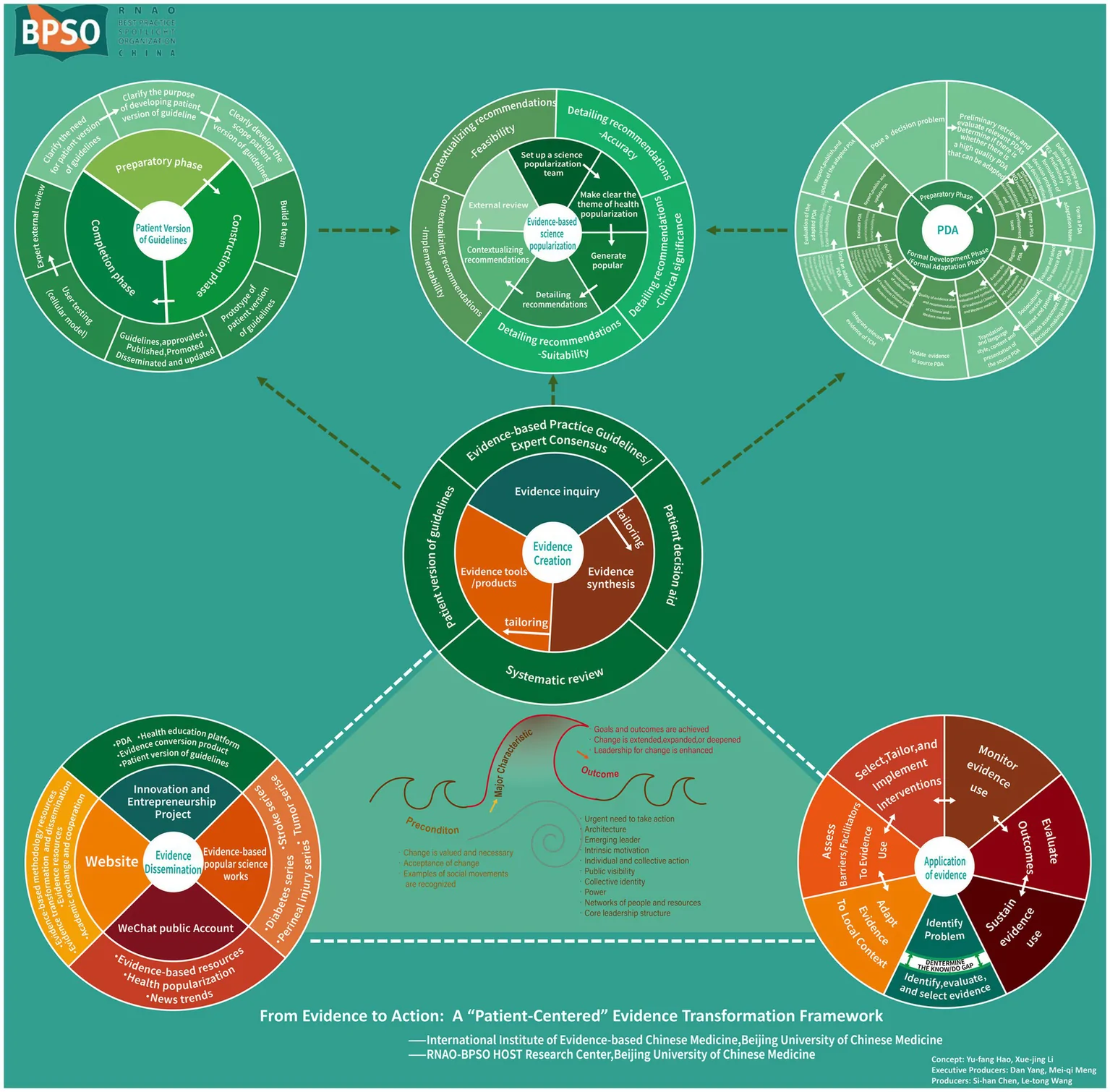
From evidence to action: a “patient-centered” evidence transformation framework. For details of each section in Figure 1, please refer to the Supplementary materials; PDA, patient decision aids.

As shown in Figure 1, this framework comprises a core component (Evidence Creation) and five functional modules: Patient Version of Guidelines, Evidence-Based Science Popularization, PDA, Evidence Dissemination, and Application of Evidence. These six components are not arranged linearly but are interconnected, collectively forming a closed-loop transformation pathway from evidence creation to patient benefit. The KTA model provides a structural framework for evidence creation, refinement, implementation, and evaluation, while the SMM serves as a driving force for collective participation, leadership development, and long-term sustainability (42, 51). Through this dual-driven mechanism, the framework transcends the traditional “guideline implementation” approach and establishes a systematic evidence co-creation model in which patients actively participate throughout the entire transformation process.

### 5.1 Evidence creation

As shown in Supplementary Figure 1, Evidence Creation serves as the core hub and foundational source of the entire framework, corresponding to the knowledge creation phase in the KTA model, and provides the scientific basis for all subsequent patient tools, communication strategies, and evidence applications. This module comprises four consecutive and iterative processes: evidence generation, evidence review, evidence synthesis, and evidence production (41, 52). The objective of this stage is to transform clinical questions into structured evidence resources through systematic searches, critical evaluations, comprehensive analyses, and adaptive applications of existing best evidence. The primary outputs of this stage include systematic reviews, evidence syntheses, expert consensus statements, and clinical practice guidelines. These outcomes not only provide professional guidance for healthcare providers but also establish a solid evidence foundation for patient-facing tools (such as patient version of guidelines and PDA) (53). Unlike traditional evidence creation approaches primarily designed for professionals, patient-centered evidence creation emphasizes the early incorporation of patients’ needs, values, and real-world clinical contexts during evidence screening and refinement. This ensures that the generated evidence possesses not only scientific rigor but also clinical relevance and patient-centered alignment.

### 5.2 Patient version of guidelines

As shown in Supplementary Figure 2, Patient Version of Guidelines serve as the primary tool for translating professional evidence into health recommendations that are easy for patients to understand, accessible, and practical to implement. Its main objectives are to enhance patients’ health literacy, strengthen their self-management capabilities, and support their active participation in healthcare decision-making (54, 55). Traditional clinical practice guidelines are primarily designed for healthcare professionals and often contain highly specialized terminology, making them difficult for patients to comprehend and apply. Patient-friendly guidelines simplify and rephrase professional recommendations in language that is easy for patients to understand, while ensuring the accuracy of evidence foundations and consistency of recommendations (56). The development process typically involves three stages: preparation phase, construction phase, and completion phase. The preparation phase clarifies development requirements, project scope, and target population; the construction phase involves forming a multidisciplinary team comprising practitioners, methodologists, and patient representatives, followed by prototype development; the completion phase includes patient testing, external expert review, dissemination of results, version updates, and long-term maintenance (57–59). This module ensures that evidence-based recommendations can be effectively integrated into patients’ daily health management within professional healthcare systems.

### 5.3 Evidence-based science popularization

As shown in Supplementary Figure 3, Evidence-Based Science Popularization serves as a bridge between professional evidence and public understanding. Its core mission is to transform complex evidence-based knowledge into easily comprehensible, accessible, and practice-oriented health education materials for patients and the general public (11, 60). Traditional health education often relies on experience-based communication approaches, which may lack scientific rigor or standardization. In contrast, the dissemination of evidence-based science ensures that all health communication content originates from validated evidence sources and is presented in clear, relevant, and patient-friendly terms (48, 61). This module aims to convert guideline recommendations and evidence summaries into practical science communication products, such as educational manuals, visual infographics, concise educational texts, and multimedia resources. The development process includes identifying patients’ core concerns, refining key recommendations, translating information into everyday language, and conducting readability and usability tests (11, 62). Through this approach, relevant evidence can be understood and applied by patients in their daily decision-making and self-care practices.

### 5.4 PDA

As shown in Supplementary Figure 4, PDA serves as a core tool for collaborative decision-making and represents one of the most direct manifestations of patient-centered evidence transformation. By presenting evidence-based treatment options, their benefits, risks, and value-sensitive trade-offs in a structured format, PDA facilitates collaborative decision-making between patients and healthcare professionals (63, 64). When multiple clinically appropriate treatment options exist and patient preferences significantly influence the final decision, SDM becomes particularly crucial. PDA helps reduce decision-making conflicts, enhance patient understanding, improve treatment adherence, and increase satisfaction with healthcare services (65, 66). The development of PDA typically involves stages such as needs assessment, option clarification, evidence integration, value clarification design, prototype testing, and iterative optimization. Unlike patient version of guidelines that primarily support holistic health management, PDA is specifically designed for preference-sensitive decisions in clinical practice (67). This module ensures that patients actively participate in treatment decisions based on scientific evidence and their personal values, rather than passively receiving care.

### 5.5 Evidence dissemination

As shown in Supplementary Figure 5, Evidence Dissemination is dedicated to expanding the reach of evidence-based practices, enhancing their visibility, and strengthening their sustainability. Through multidimensional communication strategies, it transforms isolated knowledge outcomes into widely shared professional and patient resources (68). This module encompasses professional education, patient education platforms, public health communication systems, digital information dissemination channels, and standardized evidence transformation products. Its objective is to ensure the efficient circulation of evidence across different levels of the healthcare system and its effective delivery to both professionals and patients (45, 69). Unlike traditional one-way knowledge transmission, patient-centered evidence dissemination emphasizes two-way communication, stakeholder engagement, and collaborative efforts. By incorporating SMM principles—such as collective identity, public visibility, and grassroots mobilization—evidence dissemination becomes a process of social participation rather than passive information dissemination (23, 42, 70). This approach not only enhances organizational implementation capabilities but also improves long-term patient engagement.

### 5.6 Application of evidence

As shown in Supplementary Figure 6, Application of Evidence marks the final implementation phase and ultimate objective of the entire framework. It corresponds to the action cycle of the KTA model, establishing a dynamic closed-loop pathway that translates evidence into sustainable improvements in clinical practice (3, 71). This module encompasses the adaptive adaptation of evidence to local contexts, assessment of barriers and facilitators, implementation of interventions, monitoring application, outcome evaluation, and sustainable maintenance (44, 72). The module emphasizes that application of evidence is not a one-time activity but a continuous cycle of quality improvement and practice optimization. The focus at this stage is on ensuring that evidence is effectively integrated into real-world clinical practice, rather than remaining at the level of theoretical recommendations. Successful implementation requires contextual adaptation, stakeholder collaboration, leadership support, and ongoing evaluation of outcomes (46, 73). Concurrently, this module reflects the outcome objectives of SMM: not only promoting quantifiable improvements in clinical practice and patient outcomes but also driving deeper organizational cultural transformation and strengthening leadership for long-term change (23, 74). Ultimately, the module ensures that the entire framework achieves its ultimate goal: translating high-quality evidence into tangible patient benefits and advancing sustainable improvements in healthcare services.

## 6 Proposed integrated framework

The development of a patient-centered evidence transformation framework represents not merely an extension of traditional implementation models, but rather a necessary reconstruction of the conceptual logic underlying evidence-based practice. Existing theoretical models provide critical perspectives for evidence implementation. However, these models often focus solely on specific stages of evidence transformation (e.g., evidence creation, contextual adaptation, organizational support, or patient participation in clinical decision-making) rather than treating the entire evidence lifecycle as an integrated system (3, 9, 10, 40). To address these limitations, this study proposes an integrated framework built upon the original RNAO - BPSO model. This framework combines structural rigor, contextual adaptability, collective mobilization mechanisms, and patient-centered engagement patterns, aiming to establish a sustainable pathway for translating evidence into tangible patient benefits.

### 6.1 Theoretical justification

The theoretical foundation of the proposed framework lies in the complementary strengths of existing implementation theories. The KTA model provides a systematic, iterative process for translating knowledge into practice, focusing on evidence creation, adaptive adjustments, implementation execution, process monitoring, and sustainability assurance (3, 75). While the model features a rigorous procedural framework, its coverage of emotional engagement, collective identity, and cultural transformation remains relatively limited.

SMM addresses this limitation by conceptualizing evidence implementation as a bottom-up process driven by shared values, intrinsic motivation, and collective action (36, 76). The framework emphasizes urgency, emerging leadership, grassroots participation, and public engagement. These elements are critical for achieving organizational transformation that goes beyond short-term compliance requirements (42, 77).

Therefore, the proposed integrated framework is theoretically well-grounded: it combines the procedural logic of KTA model, the mobilization power of SMM, and the patient-centered philosophy. This integration transforms evidence transformation from a single implementation event into a continuous co-constructive process involving the active participation of patients, healthcare professionals, organizations, and the healthcare system.

### 6.2 Practical necessity

The practical necessity of this comprehensive framework stems from the persistent challenges encountered in implementing real-world evidence. Despite the rapid increase in clinical practice guidelines, systematic reviews, and evidence syntheses, the process of translating evidence into routine clinical practice remains slow, fragmented, and often unsustainable (41, 78). The well-known “cognition-practice gap” continues to limit the effectiveness of evidence-based medicine systems (3, 79).

In many healthcare settings, the application of evidence remains predominantly professional-centered, with limited patient involvement in the adaptation, dissemination, and utilization of evidence. Patients are typically included only at the final stage of clinical decision-making rather than throughout the entire evidence transformation process (80). This approach diminishes the feasibility and long-term sustainability of evidence application, particularly in areas requiring active patient participation such as chronic disease management, rehabilitation therapy, and community care (2, 81). Furthermore, healthcare systems face increasingly complex challenges, including population aging, comorbidities, digital healthcare transformation, and the demand for personalized care. These challenges necessitate the adoption of flexible, culturally adaptive evidence transformation models that integrate multidisciplinary collaboration and patient autonomy in decision-making (45, 82).

This practical requirement is particularly prominent in integrated Chinese and Western nursing systems, as it necessitates the transformation of evidence across different epistemological frameworks, clinical cultures, and patient expectations. Fragmented implementation models are insufficient to address this complexity adequately. Therefore, it is imperative to establish a patient-centered integrated framework to ensure that evidence maintains both scientific rigor and contextual relevance.

### 6.3 International significance

The proposed framework holds significant international relevance, as it aligns with global trends toward patient-centered care, the establishment of evidence-based healthcare systems, and the sustainable transformation of healthcare delivery. The World Health Organization (WHO), RNAO, and multiple guideline development bodies have increasingly emphasized that patient engagement, shared decision-making, and sustainable implementation are key elements for improving healthcare quality (83, 84). The RNAO - BPSO project demonstrates that the sustainable implementation of evidence-based practices requires not only high-quality guidelines but also strong leadership, context-specific adaptations, and long-term organizational commitment (84, 85). Similarly, the WHO’s patient-centered comprehensive care framework underscores that patients should not be merely recipients of healthcare services but rather partners in the design and improvement of healthcare systems (83, 86).

The proposed framework contributes to this international discussion by constructing a model that integrates Evidence Creation, Patient Version of Guidelines, Evidence-Based Science Popularization, PDA, Evidence Dissemination, and Application of Evidence into a continuous system (42, 51). This approach provides a replicable framework for other countries facing similar challenges in evidence implementation, particularly suitable for low-and middle-income countries with limited resources—regions that require efficient and patient-centered transition pathways (5, 42, 49, 50). Furthermore, by incorporating localized adaptation and cultural response mechanisms, the framework makes significant contributions to global discussions on how implementation science can transcend universal models and support context-specific transitions (3, 9, 10, 40). It introduces a China perspective within the international field of implementation science while maintaining methodological compatibility with global evidence-based practice standards.

Therefore, the proposed integrated framework represents not only an innovative local methodology but also a significant contribution to the international advancement of patient-centered implementation science.

## 7 Applications in integrative Chinese and Western nursing

The proposed Patient-Centered Evidence Transformation Framework provides a conceptual pathway for translating evidence into clinical practice within integrative Chinese and Western nursing. Rather than viewing evidence implementation as a clinician-driven process, this framework emphasizes patients as active participants throughout the evidence lifecycle, including evidence accessibility, shared decision-making, self-management, and individualized care adaptation. Therefore, integrative Chinese and Western nursing provides an important context for demonstrating how Evidence Creation, Patient Version of Guidelines, Evidence-Based Science Popularization, PDA, Evidence Dissemination, and Application of Evidence can be interconnected to support patient-centered outcomes (3, 5, 36).

Both domestic and international studies have demonstrated that the key to successfully implementing evidence-based practices in nursing lies in the organic integration of standardized evidence-based pathways with context adaptation. At the international level, frameworks such as RNAO - BPSO emphasize guideline localization, leadership development, and continuous quality improvement (84, 87); in China, evidence-based nursing initiatives increasingly focus on localizing best practice guidelines and integrating TCM nursing interventions into evidence-based protocols (7, 88). The following section outlines several representative application areas.

Beyond its application in specific clinical scenarios, this framework provides practical guidance for all stakeholders involved in translating evidence-based practices. As summarized in Table 3 and graphical abstract (D), it offers tailored recommendations for clinical nurses, nurse educators, hospital administrators, and policymakers to promote patient-centered evidence-based practices at individual, organizational, educational, and healthcare system levels. This stakeholder-oriented approach further enhances the framework’s applicability and facilitates its integration into routine care practices and healthcare policies.

**Table 3:** Practical implications of the proposed patient-centered evidence transformation framework for key stakeholders.

Stakeholder	Framework components	Recommended actions	Expected impact
Clinical nurses	Patient version of guidelines, evidence-based science popularization, PDA, application of evidence	Deliver patient education; facilitate SDM; evaluate implementation	Improved patient engagement and adherence
Nurse educators	Evidence creation, evidence-based science popularization	Teach implementation science; develop AI-assisted learning; integrate implementation competencies	Enhanced implementation capacity
Hospital administrators	Evidence dissemination, application of evidence	Leadership support; implementation teams; quality monitoring	Sustainable evidence implementation
Policymakers	Evidence creation, evidence dissemination	Support guideline development; establish policy incentives; fund multicenter implementation	System-level evidence uptake

PDA, patient decision aids; SDM, shared decision-making; AI, artificial intelligence.

### 7.1 Stroke care

Stroke care represents an important application scenario of the Patient-Centered Evidence Transformation Framework because stroke rehabilitation requires continuous evidence adaptation, patient engagement, and long-term self-management support. Internationally, evidence-based stroke nursing emphasizes early screening for dysphagia, nutritional risk assessment, rehabilitation training, aspiration prevention, and continuity of care (89). International guideline systems, including RNAO recommendations, highlight standardized swallowing assessments and multidisciplinary rehabilitation pathways to improve patient safety and functional outcomes (84, 89).

Within the proposed framework, these evidence resources correspond primarily to Evidence Creation and Patient Version of Guidelines modules, in which complex clinical recommendations are transformed into accessible information that enables patients and families to participate in rehabilitation decision-making. Domestic practices have further incorporated TCM nursing approaches, including acupressure, acupoint stimulation, rehabilitation training, and syndrome-based nursing interventions, into post-stroke dysphagia, spasticity, and motor dysfunction management (90–92). These localized adaptations reflect the framework’s emphasis on contextual modification and patient-centered application of evidence.

Our team’s evidence-based practice project on stroke-related dysphagia management demonstrates how standardized evidence screening can be combined with localized TCM nursing interventions to improve early identification, reduce aspiration risk, and enhance rehabilitation continuity (93–95). Importantly, this process highlights that evidence transformation is not only the adoption of clinical recommendations but also the adaptation of evidence into understandable, culturally appropriate, and actionable care strategies for patients and caregivers.

### 7.2 Diabetic foot management

Diabetic foot management provides another example of how the Patient-Centered Evidence Transformation Framework facilitates the transition from evidence generation to patient empowerment. International evidence supports risk stratification, regular foot assessment, wound management, glycemic control, and patient self-management education as core components of prevention and management (96). The International Working Group on the Diabetic Foot (IWGDF) has developed standardized recommendations to guide evidence-based diabetic foot care (97).

According to the proposed framework, these recommendations should not remain limited to healthcare professionals but should be transformed through Evidence-Based Science Popularization, PDA, and Evidence Dissemination modules to improve patients’ understanding of risk factors, daily foot care behaviors, and treatment choices. In China, diabetic foot nursing has increasingly incorporated TCM-based approaches, including external therapies, acupressure, foot care education, and individualized lifestyle management (98). Evidence-based nursing protocols further demonstrate that continuous education and supportive nursing interventions improve adherence and reduce ulcer recurrence (99).

Compared with international approaches emphasizing standardized risk management, Chinese integrative nursing practices place greater emphasis on family involvement, behavioral adaptation, and long-term self-management. These characteristics align with the patient-centered framework by recognizing patients and families as active partners rather than passive recipients of evidence-based recommendations.

### 7.3 Pelvic floor rehabilitation

Postpartum pelvic floor rehabilitation illustrates the importance of integrating evidence-based interventions with individualized and culturally responsive care. International guidelines emphasize early screening, pelvic floor muscle training, urinary function assessment, and long-term follow-up to prevent urinary incontinence and pelvic organ prolapse (100).

Within the Patient-Centered Evidence Transformation Framework, pelvic floor rehabilitation represents an application pathway in which evidence is translated into patient-friendly education, SDM tools, and individualized rehabilitation plans. Domestic clinical practice has incorporated TCM rehabilitation approaches, including acupoint stimulation, moxibustion, postpartum health education, and syndrome-based nursing strategies (101). Evidence-based protocols suggest that combining standardized pelvic floor muscle training with individualized education and supportive interventions may improve adherence and rehabilitation outcomes (102, 103).

This field demonstrates how evidence transformation can bridge international standardized rehabilitation principles with culturally adapted postpartum care. Through patient education, empowerment, and continuous engagement, integrative Chinese and Western nursing extends evidence implementation from clinical intervention toward sustained patient self-management.

### 7.4 De-implementation

De-implementation represents an essential but often overlooked component of Patient-Centered Evidence Transformation Framework. While traditional implementation science has mainly focused on introducing new evidence-based interventions, sustainable healthcare improvement also requires identifying and reducing unnecessary, ineffective, or potentially harmful practices (104). International research increasingly recognizes de-implementation as a strategy to improve healthcare quality, patient safety, and resource allocation (105).

Within the proposed framework, de-implementation extends beyond removing outdated practices; it requires transparent communication with patients, consideration of patient preferences, and SDM regarding changes in care pathways. In nursing practice, this may include discontinuing unnecessary routine procedures, replacing unsupported rehabilitation approaches, and introducing evidence-based alternatives while maintaining patient trust and engagement (106).

In the context of integrative Chinese and Western nursing, de-implementation is particularly important because some traditional practices may continue despite insufficient evidence. However, evidence transformation should not simply eliminate traditional approaches; rather, it should evaluate their effectiveness, safety, and acceptability from both scientific and patient perspectives (107, 108). Therefore, Patient-Centered Evidence Transformation Framework positions de-implementation as a collaborative process in which clinicians and patients jointly determine whether an intervention provides meaningful benefit, ensuring that quality improvement is achieved without compromising patient values or cultural needs.

This perspective expands conventional de-implementation concepts by incorporating patient empowerment, cultural sensitivity, and SDM as essential elements of evidence transformation.

## 8 Future research and implications

As shown in graphical abstract (C,E), the future research of patient-centered evidence transformation should move beyond traditional evidence implementation models toward intelligent, digital, and globally adaptable systems (109). With healthcare increasingly transitioning toward precision medicine, digital health, and patient-centered care models, evidence transformation frameworks must continuously evolve to support real-time decision-making, sustained patient engagement, and cross-system sustainability (16, 110). In particular, the integration of elements such as Artificial Intelligence (AI), digital PDA, multicenter validation, global adaptation, and cross-cultural transferability will be key drivers for advancing the scientific implementation of integrated Chinese and Western nursing.

### 8.1 AI

AI is poised to fundamentally transform the evidence transformation process by enhancing the efficiency, accuracy, and scalability of evidence creation, dissemination, and application. Traditional evidence transformation often relies on labor-intensive systematic reviews, manual evidence evaluation, and delays in clinical implementation (111). AI technologies—including natural language processing, machine learning, clinical decision support systems, and large language models—can significantly accelerate evidence integration, identify gaps in clinical practice, and support the generation of personalized treatment recommendations (112, 113).

In nursing practice, AI can assist in real-time risk screening, symptom monitoring, development of predictive intervention protocols, and automated evidence updates. For example, AI-assisted clinical decision support systems enhance early warning capabilities for stroke-related dysphagia in older patients, diabetic foot risk stratification, and frailty status (114). During guideline development, machine-assisted literature screening and evidence mapping techniques simultaneously improve methodological efficiency and shorten update cycles (115).

However, the integration of AI must also address data quality, algorithmic transparency, ethical governance, and patient trust. Therefore, future frameworks should not position AI as a substitute for patient-centered care, but rather as a supportive tool to enhance evidence-based responsiveness and facilitate the implementation of personalized treatment plans (116).

### 8.2 Digital PDA

The development of digital PDA represents one of the most critical research for enhancing patient engagement in evidence transformation (117). Traditional paper-based PDA tools often suffer from poor accessibility, difficulty in updating, and limited applicability in clinical practice. In contrast, digital PDA platforms delivered through mobile applications, hospital information systems, web interfaces, and WeChat mini-programs enable more interactive and personalized evidence-based decision support, along with continuous updates (118).

Digital PDA can provide multimedia explanations of treatment plans, visual risk assessments, value clarification exercises, and personalized follow-up support, thereby enhancing decision-making quality and reducing information asymmetry between clinical practitioners and patients (119). In the fields of chronic disease management, rehabilitation care, and oncology diagnosis and treatment, digital PDA also offers continuous decision support outside hospital settings (120, 121).

Future developments should prioritize integrating digital PDA into clinical workflows, ensuring their interoperability with electronic health records and enhancing their usability for older patients and individuals with lower health literacy (122). Digital tools should enhance rather than replace the interaction between patients and healthcare professionals.

### 8.3 Multicenter validation

To enhance scientific rigor and implementation credibility, multicenter validation should serve as the core strategy for evaluating patient-centered evidence-based transformation frameworks. Currently, many evidence-based nursing initiatives remain confined to single-center implementation studies, which limits the generalizability and external validity of their findings (123).

Multicenter validation enables researchers to assess whether the evidence-based intervention pathway remains effective across different hospital levels, healthcare regions, nursing teams, and patient populations (124). This is particularly critical for complex interventions such as integrated Traditional Chinese Medicine and Western Medicine nursing care, as contextual variations can significantly impact their implementation outcomes (125).

Future research should establish standardized outcome indicators that encompass patient outcomes, implementation consistency, leadership engagement, cost-effectiveness, and sustainability (126). The adoption of a mixed methods design combining quantitative outcomes with qualitative implementation experiences will further enhance the interpretative power of multicenter studies (127). Only through large-scale multicenter validation can this framework evolve from localized innovations into a widely recognized implementation model.

### 8.4 Global adaptation

Global-level adaptive adjustments are crucial for enhancing the international impact of patient-centered evidence transformation frameworks. Although the implementation of science has advanced rapidly in high-income countries, significant gaps remain in adapting evidence transformation models to diverse healthcare systems, resource conditions, and cultural contexts (83, 111).

The proposed framework—developed based on KTA, SMM, RNAO - BPSO, and patient-centered nursing principles—is methodologically highly compatible with international implementation science. However, its practical application requires adaptation to local policy environments, human resource capacities, digital infrastructure, and patient expectations (87, 128). For instance, in low-and middle-income countries, implementation efforts may prioritize resource efficiency, community engagement, and streamlined decision-support processes (129). In highly digitized systems, AI-powered decision-support tools may serve as the primary driver of implementation success (130).

Future research should explore how to localize this framework while preserving its core theoretical integrity, thereby supporting both methodological standardization and flexible contextual adaptation.

### 8.5 Cross-cultural transferability

Cross-cultural transferability is particularly crucial for evidence transformation in integrated Chinese and Western nursing, as the application of evidence is significantly influenced by values, communication styles, family structures, and health beliefs (2, 131).

SDM, patient version of guidelines development, and the application of PDA may vary across different cultural contexts. For example, Western healthcare systems typically emphasize individual autonomy, whereas Chinese clinical practice tends to favor family-centered decision-making models and collective accountability mechanisms (47, 132). Similarly, perceptions of traditional medicine, trust in professional authorities, and expectations for self-management also exhibit significant differences across various medical cultures (133).

Therefore, evidence transformation tools cannot be directly applied without cultural adaptation. The patient versions of guidelines, science popularization, and the structure of PDA systems all require redesign based on linguistic habits, decision-making norms, and societal values (10). Future research should prioritize cross-cultural adaptation approaches, international comparative validation, and multilingual communication strategies (134). This will enable patient-centered evidence transformation to transcend localized successes and make substantial contributions to global scientific implementation.

Ultimately, the future of evidence transformation lies in building intelligent, digital, scalable, and culturally adaptive systems that enable patients to become genuine co-creators of healthcare improvement, rather than passive recipients of evidence.

## 9 Limitations

Although this paper has achieved significant results at the theoretical level, it still faces three major limitations: (1) Further empirical research is required to validate this framework. (2) The narrative review may suffer from selection bias, as the included literature is influenced by authors’ subjective factors. (3) Since the study was conducted in China, applying this framework across different national healthcare systems presents challenges, thereby affecting its generalizability.

## 10 Conclusion

This review proposes a Patient-Centered Evidence Transformation Framework by integrating the KTA model and the SMM to address the persistent evidence–practice gap in nursing. The framework consists of six interconnected components—Evidence Creation, Patient Version of Guidelines, Evidence-Based Science Popularization, PDA, Evidence Dissemination, and Application of Evidence—which together provide a continuous pathway from evidence generation to patient benefit.

By placing patients at the center of the evidence transformation process, the proposed framework extends existing implementation theories and offers a practical approach for improving evidence uptake, SDM, and the sustainability of evidence-based practice in integrative Chinese and Western medicine nursing. It also provides a conceptual reference for promoting patient-centered implementation in broader healthcare settings.

Future research should focus on validating the framework in diverse clinical contexts, evaluating its implementation effectiveness, and refining its application through multidisciplinary collaboration and continuous evidence generation.
