# Supplementary material for: New perspectives from evidence to action — patient-centered evidence transformation in evidence-based nursing: a narrative review
Source: Front Public Health. 2026 Aug 28;14:1877125. doi: 10.3389/fpubh.2026.1877125 (PMC13563161; doi:10.3389/fpubh.2026.1877125)
Supplement: Supplementary file 1 [file Data_Sheet_1.DOCX]

Scale for the Assessment of Narrative Review Articles – SANRA

Please rate the quality of the narrative review article in question, using categories 0–2 on the following scale. For each aspect of quality, please choose the option which best fits your evaluation, using categories 0 and 2 freely to imply general low and high quality. These are not intended to imply the worst or best imaginable quality.

**1) Justification of the article’s importance for the readership**

The importance is not justified. 0

The importance is alluded to, but not explicitly justified. 1	2

The importance is explicitly justified. 2

*The manuscript clearly identifies the persistent evidence–practice gap in evidence-based nursing and highlights the need for patient-centered evidence transformation, especially in integrative Chinese and Western medicine nursing. The importance for readers is explicitly justified in both the Introduction and Abstract.*

**2) Statement of concrete aims or formulation of questions**

No aims or questions are formulated. 0

Aims are formulated generally but not concretely or in terms of clear questions. 1	2

One or more concrete aims or questions are formulated. 2

*The objective is clearly stated: to explore patient-centered evidence transformation pathways by integrating the Knowledge to Action Model and the Social Movement Model and to propose an integrated framework. The aim is specific and consistent throughout the manuscript.*

**3) Description of the literature search**

The search strategy is not presented. 0

The literature search is described briefly. 1	2

The literature search is described in detail, including search terms and inclusion criteria. 2

*The manuscript states that a narrative review approach was adopted and summarizes the theoretical analysis process, but detailed search terms, databases, and inclusion criteria are not fully described. This meets brief description rather than detailed reporting.*

**4) Referencing**

Key statements are not supported by references. 0

The referencing of key statements is inconsistent. 1	2

Key statements are supported by references. 2

*Key theoretical statements, model comparisons, and practical applications are consistently supported by references. Core arguments related to Knowledge to Action model Model, Social Movement Model, Registered Nurses’ Association of Ontario - Best Practice Spotlight Organizations, Patient Decision Aid, and implementation science are well referenced throughout the manuscript.*

**5) Scientific reasoning**

*(e.g., incorporation of appropriate evidence, such as RCTs in clinical medicine)*

The article’s point is not based on appropriate arguments. 0

Appropriate evidence is introduced selectively. 1	2

Appropriate evidence is generally present. 2

*The manuscript demonstrates strong scientific reasoning by integrating multiple implementation science theories (Knowledge-to-Action Model, Social Movement Model, Promoting Action on Research Implementation in Health Services Framework, Consolidated Framework for Implementation Research, Shared Decision-Making Model) and using appropriate literature to support conceptual arguments and practical implications. Evidence is appropriately synthesized rather than selectively introduced.*

**6) Appropriate presentation of data**

*(e.g., absolute vs relative risk; effect sizes without confidence intervals)*

Data are presented inadequately. 0

Data are often not presented in the most appropriate way. 1	2

Relevant outcome data are generally presented appropriately. 2

*As a narrative review, the manuscript appropriately presents conceptual evidence, implementation pathways, and comparative findings without misuse of quantitative outcome reporting. Arguments and examples are presented in a clear and suitable format for this article type.*

**Sumscore	11**

SANRA – explanations and instructions

This scale is intended to help editors assess the quality of a narrative review article based on formal criteria accessible to the reader. It cannot cover other elements of editorial decision making such as degree of originality, topicality, conflicts of interest or the plausibi- lity, correctness or completeness of the content itself. SANRA is an instrument for editors, authors, and reviewers evaluating individual manuscripts. It may also help editors to document average manuscript quality within their journal and researchers to document the manuscript quality, for example in peer review research. Using only three scoring options, 0, 1 and 2, SANRA is intended to provide a swift and pragmatic sum score for quality, for everyday use with real manuscripts, in a field where established quality standards have previously been lacking. It is not designed as an exact measurement of the quality of all theoretically possible manuscripts. For this reason, the extreme values (0 and 2) should be used relatively freely and not reserved only for perfect or hopeless articles.

We recommend that users test-rate a few manuscripts to familiarize themselves with the scale, before using it on the intended group of manuscripts. Ratings should assess the totality of a manuscript, including the abstract. The following comments clarify how each question is designed to be used.

**Item 1 – Justification of the article’s importance for the readership**

Justification of importance for the readership must be seen in the context of each journal’s readership.

Consider how well the manuscript outlines the clinical problem and highlights unanswered questions or evidence gaps – thoroughly (2), superficially (1), or not at all (0).

**Item 2 – Statement of concrete/specific aims or formulation of questions**

A good paper will propose one or more specific aims or questions which will be dealt with or topics which will be reviewed. Please rate whether this has been done thoroughly and clearly (2), vaguely or unclearly (1), or not at all (0).

**Item 3 – Description of the literature search**

A convincing narrative review will be transparent about the sources of information on which the text is based. Please rate the degree to which you think this has been achieved. To achieve a rating of 2, it is not necessary to describe the literature search in as much de- tail as for a systematic review (searching multiple databases, including exact descriptions of search history, flowcharts, etc.), but it is necessary to specify search terms, and the types of literature included. A manuscript which only refers briefly to its literature search would score 1, while one not mentioning its methods would score 0.

**Item 4 – Referencing**

No manuscript references all statements. However, those that are essential for the arguments of the manuscript – “key statements” – should be backed by references in all or almost all cases. Exceptions could reasonably be made for rating purposes where a key

statement has uncontroversial face-validity, such as “Diabetes is among the commonest causes of chronic morbidity worldwide.” Please rate the completeness of referencing: for most or all relevant key statements (2), inconsistently (1), sporadically (0).

**Item 5 – Scientific reasoning**

The item describes the quality of the scientific point made. A convincing narrative review presents evidence for key arguments. It should mention study design (randomized controlled trial, qualitative study, etc), and where available, levels of evidence.

Please rate whether you feel this has been done thoroughly (2), superficially (1), or hardly at all (0). Unlike item 6, which is

concerned with the selection and presentation of concrete outcome data, this item relates to the use of evidence and of types of evidence in the manuscript’s arguments.

**Item 6 – Appropriate presentation of data:**

This item describes the correct presentation of data central to the article’s argument. Which data are considered relevant varies from field to field. In some areas relevant data would be absolute rather than relative risks or clinical versus surrogate or intermediate end- points. These outcomes must be presented correctly. For example, it is appropriate that effect sizes are accompanied by confidence intervals. Please rate how far the paper achieves this – thoroughgoingly (2), partially (1), or hardly at all (0). Unlike item 5, which

relates to the use of evidence and of types of evidence in the manuscript’s arguments, this item is concerned with the selection and presentation of concrete outcome data.

**Reference**

Baethge C, Goldbeck-Wood S, Mertens S: SANRA—a scale for the quality assessment of narrative review articles. Research Integrity and Peer Review (2019) 4:5

<https://doi.org/10.1186/s41073-019-0064-8>
