# Supplementary material for: New perspectives from evidence to action — patient-centered evidence transformation in evidence-based nursing: a narrative review
Source: Front Public Health. 2026 Aug 28;14:1877125. doi: 10.3389/fpubh.2026.1877125 (PMC13563161; doi:10.3389/fpubh.2026.1877125)
Supplement: Supplementary file 2 [file Data_Sheet_2.DOCX]

**Supplementary Materials**

**1. Methodology**

*1.1 Search Strategy*

Two reviewers (Zhe-Hao Hu and Liu Han) independently used EndNote 21 to screen article titles and abstracts. After completing the abstract screening, they downloaded the full-text articles for detailed review.

*1.2 Databases*

PubMed, Embase, Cochrane Library, Web of Science, Scopus, Chinese National Knowledge Infrastructure, Chinese Scientific Journals Database, Wanfang Database, Chinese BioMedical Literature Database.

*1.3 Keywords*

Under the "Narrative Review" condition, the Keywords for each section are distinct. (Shown on Table 1.)

**Table 1.**

Search terms used for literature identification

| **Concept domain** | **Search keywords** |
| --- | --- |
| Evidence transformation | "evidence transformation" OR "evidence translation" OR "evidence implementation" OR "evidence-based practice" OR "knowledge translation" OR "knowledge utilization" |
| Nursing practice | nursing OR nursing practice OR evidence-based nursing OR clinical nursing OR nursing intervention |
| Implementation science | implementation science OR implementation research OR implementation framework OR implementation model OR knowledge-to-action OR KTA OR PARIHS OR CFIR |
| Patient-centered approach | patient-centered care OR person-centered care OR patient engagement OR patient participation OR patient empowerment OR shared decision-making |
| Patient-oriented evidence tools | patient decision aid OR PDA OR patient version of guidelines OR patient-friendly guideline OR health literacy |
| Evidence dissemination | evidence dissemination OR knowledge dissemination OR evidence communication OR science popularization |
| Integrative Chinese and Western nursing | integrative nursing OR integrated Chinese and Western medicine OR traditional Chinese medicine nursing OR TCM nursing OR complementary medicine nursing |

*1.4 Inclusion and Exclusion Criteria*

Inclusion criteria: (1) Commonly used evidence-based nursing transformation models; (2) Literature supporting the "Patient-Centered" Evidence Transformation Framework; (3) International literature relevant to our research focus; (4) Literature that supports the methodology and perspectives of this study.

Exclusion criteria: (1) Literature that cannot be downloaded in full; (2) Outdated literature with updated versions.

*1.5 Time Period*

From database creation to May 1, 2026.

*1.6 Method of Selecting Literature*

In the event of a disagreement between two reviewers (Zhe-Hao Hu and Liu Han), a discussion shall be conducted first; if the issue remains unresolved, either the literature may be excluded from inclusion or the corresponding author of the relevant article may be consulted for clarification. After performing fuzzy search, titles and abstracts are reviewed; articles meeting the inclusion criteria are downloaded and read in full, followed by comparative analysis to select those most consistent with the study's objectives.

**2. Elements in Figure 2.**

Figure 1. Evidence Creation.

Figure 2. Patient Version of Guidelines.

Figure 3. Evidence-Based Science Popularization.

Figure 4. PDA.

Figure 5. Evidence Dissemination.

Figure 6. Application of Evidence.

Figure 7. SMM.
